# *Kupeantha* (Coffeeae, Rubiaceae), a new genus from Cameroon and Equatorial Guinea

**DOI:** 10.1371/journal.pone.0199324

**Published:** 2018-06-26

**Authors:** Martin Cheek, Maria G. Alvarez-Aguirre, Aurélie Grall, Bonaventure Sonké, Melanie-Jayne R. Howes, Isabel Larridon

**Affiliations:** 1 Herbarium, Royal Botanic Gardens, Kew, Richmond, Surrey, United Kingdom; 2 University of Yaoundé I, Higher Teacher’s Training College, Plant Systematic and Ecology Laboratory, Yaoundé, Cameroon; 3 Université Libre de Bruxelles, Herbarium et Bibliothèque de Botanique africaine, Brussels, Belgium; 4 Missouri Botanical Garden, St. Louis, Missouri, United States of America; 5 Ghent University, Department of Biology, Research Group Spermatophytes, Campus Ledeganck, Ghent, Belgium; University of Florida, UNITED STATES

## Abstract

Two new coffee relatives (tribe Coffeeae, Rubiaceae), discovered during botanical expeditions to Cameroon, are examined for generic placement, and the placement of three previously known species (*Argocoffeopsis fosimondi*, *A*. *spathulata* and *Calycosiphonia pentamera*) is reinvestigated using plastid sequence (*accD-psa1*, *rpl16*, *trnL-F*) and morphological data. Seed biochemistry of the new species and pollen micromorphology (only one of the two species) are also studied. Based on the plastid sequence data, the new taxa are nested in a well-supported monophyletic group that includes *Argocoffeopsis* and *Calycosiphonia*. Within this clade, three well-supported subclades are recovered that are morphologically easy to diagnose: (1) *Calycosiphonia* (excluding *C*. *pentamera*), (2) *Argocoffeopsis* (excluding *A*. *fosimondi* and *A*. *spathulata*), and (3) a clade including the above excluded species, in addition to the new species. Based on the results, *Kupeantha*, a new genus of five species, is described, including two new Critically Endangered taxa from the Highlands of Cameroon: *Kupeantha ebo* and *K*. *kupensis*. Phytochemical analysis of *Kupeantha* seeds reveals compounds assigned as hydroxycinnamic acid derivatives, amino acids and *ent*-kaurane diterpenoids; caffeine was not detected. *Kupeantha* is the first new genus described in tribe Coffeeae in 40 years.

## Introduction

Distribution data, although important to ensure effective conservation management and sustainable utilisation of natural resources [1], is lacking for many organisms in tropical Africa, including vascular plants necessitating renewed efforts for botanical exploration of the region [2]. Based on the RAINBIO dataset containing 600,000+ georeferenced records representing 22,000+ plant species, priority target areas for future sampling efforts were proposed in Tanzania, Atlantic Central Africa and West Africa [2]. One of the areas, which remained underexplored until recently, is Cameroon in West Central Africa. During the 1990s, the Royal Botanic Gardens, Kew and partners in Cameroon began a series of botanical expeditions to improve fundamental knowledge of plant species diversity and conservation management in western Cameroon and adjoining regions [3]. At that time, SW Cameroon was recorded as having the highest plant species diversity in Tropical Africa per degree square [4], with many species thought to be at risk of extinction. This was later confirmed on publication of the Red Data Book of the Flowering Plants of Cameroon [5], which showed that the highest concentrations of threatened plant species occurred in SW Cameroon.

Locations for botanical expeditions were selected in areas of intact natural vegetation considered of importance by local conservation NGOs that hosted the surveys. With the support of volunteers from the Earthwatch Institute, botanists collected 19,500+ herbarium specimens [6], resulting in the discovery of 100+ species new to science, most of which are threatened with extinction, e.g., *Ancistrocladus grandiflorus* Cheek [7] and *Impatiens etindensis* Cheek & Eb.Fisch. [8], and included two new genera, *Korupodendron* Litt & Cheek (Vochysiaceae) [9] and *Kupea* Cheek & S.A.Williams (Triuridaceae) [10]. Many novel Rubiaceae were also discovered [11–16].

In Mt Kupe, in the Bakossi tribal area, specimens of a cloud forest tree species resembling *Calycosiphonia* Pierre ex Robbr. (Rubiaceae) were collected in 1995, but these had several characters not seen in that genus. Based on morphology, it was suggested as a possible new genus by Bridson (pers. comm. to Cheek), but designated as *Calycosiphonia* sp. A [6]. Independently, in 2005 and 2006, a series of botanical expeditions focussed on Rubiaceae were led in South Cameroon by Sonké. These resulted in further discoveries of new taxa [17–20]. Still, the study of the family Rubiaceae in Cameroon remains far from complete. Among the collections made were numerous specimens of two species similar to *Calycosiphonia* sp. A, one with spathulate leaf tips (e.g., *Sonké & Nguembou 3783*), the other drying black (e.g., *Sonké & Djuikouo 4320*). Specimen *Sonké & Nguembou 3783* was included in the seminal study of tribe Coffeeae [21]. In the result of the combined analysis of the molecular and morphological data sets, a clade containing *Argocoffeopsis* Lebrun, *Calycosiphonia* and ‘*Calycosiphonia cf*.’ (*Sonké & Nguembou 3783*) received strong bootstrap support (BS = 99) and a high Bremer (decay) value (b = 9). Monophyly was retrieved for *Calycosiphonia* (BS = 85; b = 3) and *Argocoffeopsis* (BS = 81; b = 2), but *Calycosiphonia cf*. was placed in an unresolved position in a polytomy with the genera *Argocoffeopsis* and *Calycosiphonia* [21]. It was suggested that ‘*Calycosiphonia cf*.’ might represent an additional genus in the tribe, but that further material and morphological investigations were required [21]. Prior to allocating ‘*Calycosiphonia cf*.’ to a genus, [22] reviewed the morphological features of *Argocoffeopsis* and *Calycosiphonia*, and concluded that there was “no logic in describing *Calycosiphonia cf*. in a new genus, as apart from the size and shape of its fruit, it shows no morphological differences when compared with either *Argocoffeopsis* or *Calycosiphonia*”. They described the taxon represented by *Sonké & Nguembou 3783* as *Argocoffeopsis spathulata* A.P. Davis & Sonké [22]. The second species, represented by *Sonké & Djuikouo 4320*, was published as *Calycosiphonia pentamera* Sonké & Robbr., which necessitated broadening the generic concept [23]. Meanwhile, continued botanical expeditions in West Cameroon, this time led by Tchiengué in the cloud forest of the Lebialem Highlands in 2005–2006, led to the discovery of a fourth species of clear affinity with *Calycosiphonia* sp. A and *Argocoffeopsis spathulata*. Following [22] on generic placement, this Critically Endangered, attractive, flowering shrub was published as *Argocoffeopsis fosimondi* Tchiengué & Cheek [24]. A fifth species of ‘*Calycosiphonia cf*.’ was discovered in the proposed Ebo National Park of Cameroon’s Littoral Region with records in 2007 (*Fenton 134*) and 2015 (*Alvarez 11*).

The goal of this study is to use morphological and plastid sequence data to (1) place the newly discovered species from Mt Kupe and Ebo; and (2) reassess the generic placement of the recently described *Argocoffeopsis fosimondi*, *A*. *spathulata* and *Calycosiphonia pentamera*. Therefore, we aim at providing evidence for the delimitation of the new taxa and by publishing them formally in accordance with the *International Code of Nomenclature* (ICN) [25], we hope to draw attention to their conservation. Furthermore, high-resolution LC-UV-MS/MS seed analyses of the new species was carried out in order to contribute knowledge of the seed chemistry of the genera in the coffee tribe, the chemotaxonomic significance of the chemical constituents, and their distribution across the group. While the chemistry of the genus *Coffea* L. has been extensively investigated [26–28], there is a lack of knowledge about the chemistry of other genera, with the exception of species currently placed in *Diplospora* DC. (previously assigned to *Tricalysia* A.Rich ex DC) [27, 29–32].

## Material and methods

### Ethical statement

This study is based mainly on herbarium specimens and field observations made in Cameroon during a series of botanical surveys beginning in 1991. These surveys were mainly led by the first author. So far, they have resulted in 52,450 specimens being studied at K and YA, of which 37,850 were newly collected. Data are stored in the Kew-Cameroon specimen Access database (Gosline, p. 11 in Cheek et al. 2004). The top set of specimens was initially deposited at SCA, and later at YA, and duplicates sent to K. The fieldwork was approved by the Institutional Review Board of the Royal Botanic Gardens, Kew, named the Overseas Fieldwork Committee (OFC), under permit number 807. The most recent official invitation to carry out research on the flora and vegetation of Cameroon has reference number 050/IRAD/DG/CRRA-NK/SSRB-HN/09/2016 and it was issued under the terms of the 5-year Memorandum of Collaboration between the Institute for Research in Agricultural Development (IRAD)-Herbier National du Cameroun and the Royal Botanic Gardens, Kew, signed 5^th^ Sept 2014.

### Taxon sampling and study area

Field collections were undertaken in the Mt Kupe area (SW Region, Cameroon) between June 1995 and October 2009, and the Ebo area (Littoral Region, Cameroon) between 2007 and 2015. Herbarium material and/or digitised specimens of *Argocoffeopsis*, *Belonophora* Hook.f., *Calycosiphonia*, *Coffea*, *Diplospora*, *Discospermum* Dalzell, *Empogona* Hook.f., *Sericanthe* Robbr., *Tricalysia* and *Xantonnea* Pierre ex Pit. was consulted at BR, BRLU, K, MO, P, SCA, US, WAG and YA [33]. All specimens cited have been seen, unless otherwise indicated. Total sampling for the molecular phylogenetic study comprises 38 accessions, including 2/3 of *Calycosiphonia* species (DNA could not be extracted from material of *C*. *pentamera* and, therefore, its placement was inferred from morphological characters only), 6/10 of *Argocoffeopsis* species, and two species new to science. The samples with species names, voucher information, origin, and GenBank accession numbers, are given in S1 Appendix.

### Phylogenetic analyses

In this study, previously published sequence data were used [21, 34] and supplemented with new sequences from selected plastid regions (*accD-psa1* intergenic spacer (IGS), *rpl16* intron, and *trnL-F* intron & *trnL-F* IGS) (S1 Appendix). The DNA extraction protocol and material and methods for amplification and sequencing followed [21].

Sequences were assembled and edited in Geneious R8 (http://www.geneious.com) [35], aligned using MAFFT 7 [36, 37], and subsequently, alignments were adjusted manually in PhyDE 0.9971 [38]. The concatenated alignment used in the phylogenetic analyses is provided in S1 File.

The matrices of the three chloroplast regions were concatenated for the downstream analyses. PartitionFinder 2.1.1 [39] was used to determine an appropriate data-partitioning scheme from potential partitions that were defined *a priori* (each marker treated as a separate partition), as well as the best-fitting model of molecular evolution for each partition, using the Bayesian Information Criterion. The optimal data-partitioning scheme was *accD-psa1*+*rpl16* and *trnL-F*, and the GTR+I+Γ (invgamma) model of sequence evolution was determined to be the best-fitting model for the *accD-psa1*+*rpl16* partition, while the GTR+Γ (gamma) model of sequence evolution was determined to be the best-fitting model for the *trnL-F* partition in the concatenated data set.

Maximum likelihood (ML) analyses of the optimally partitioned data were performed using RAxML 8.2.10 [40]. The search for an optimal ML tree was combined with a rapid bootstrap analysis of 1000 replicates. Partitioned analyses were conducted using Bayesian Inference (BI) in MrBayes 3.2.6 [41]. The parameters of each of the partitions were the same as in the ML analysis. Rate heterogeneity, base frequencies, and substitution rates across partitions were unlinked. The analysis was allowed to run for 100 million generations across four independent runs with four chains each, sampling every 10000 generations. Convergence, associated likelihood values, effective sample size (ESS) values and burn-in values of the different runs were verified with Tracer 1.5 [42]. The first 25% of the trees from all runs were excluded as burn-in before making a majority-rule consensus of the 30000 posterior distribution trees using the “sumt” function. All phylogenetic analyses were run using the CIPRES portal (http://www.phylo.org/) [43]. Trees were drawn using TreeGraph2 [44].

### Morphology, palynology, distribution and conservation

Measurements, colours and other details given in the descriptions of the new species *Kupeantha ebo* and *K*. *kupensis* are based on living material, spirit, and herbarium specimens, and data and photographs derived from field notes. Pollen samples were collected only from *Kupeantha kupensis* (*Cheek 7882* K). Whole, unacetolysed anthers were placed on a stub using double-sided tape and sputter-coated with platinum in a Quorom Q150T coater for 30 seconds and examined in a Hitatchi 54700 scanning electron microscope at an acceleration voltage of 4kV.

The conservation status was assessed using the IUCN Red List Category criteria [45]. The distribution of the species was mapped using SimpleMappr [46].

### Nomenclature

The new taxon names generated as part of this study satisfy the requirements of the International Code of Nomenclature for algae, fungi, and plants, and are hereby effectively published. In addition, they have been submitted to The International Plant Names Index (IPNI), from where they will be made available to the Global Names Index (http://gni.globalnames.org/). The IPNI LSIDs will resolve and the associated information viewed through any standard web browser by appending the LSID contained in this publication to the prefix http://ipni.org/. The online version of this work is archived and available from the following digital repositories: PubMed Central, LOCKSS, and Ghent University Academic Bibliography.

### Phytochemical analysis

The extracts were prepared by extracting ground seed material from the newly described species *Kupeantha kupensis* (one accession) and *Kupeantha ebo* (two accessions). There was insufficient or no material available for the other species. Extracts were made in 80% aqueous methanol (100 mg/ml) for 24 h, prior to centrifugation. The supernatants were then subjected to LC–UV–MS/MS analysis. Analyses were performed on a Thermo Scientific system consisting of an ‘Accela’ U-HPLC unit with a photodiode array detector and an ‘LTQ Orbitrap XL’ mass spectrometer fitted with an electrospray source (Thermo Scientific, Waltham, MA, USA). Chromatography was performed on 5 μl sample injections onto a 150 mm x 3 mm, 3 μm Luna C-18 column (Phenomenex, Torrance, CA, USA) using the following 400μl/min mobile phase gradient of H_2_O/CH_3_OH/CH_3_CN +1% HCOOH: 90:0:10 (0 min), 90:0:10 (5 min), 0:90:10 (60 min), 0:90:10 (65 min), 90:0:10 (67 min), 90:0:10 (70 min) followed by return to start conditions and equilibration in start conditions for 5 min before the next injection. The ESI source was operated with polarity switching and the mass spectrometer was set to record high resolution (30 k resolution) MS1 spectra (*m/z* 125–2000) in positive mode using the orbitrap and low resolution MS1 spectra (*m/z* 125–2000) in negative mode and data dependent MS2 and MS3 spectra in both modes using the linear ion trap. Detected compounds were assigned by comparison of accurate mass data (based on ppm), and by available MS/MS data, with reference to the published compound assignment system [47] and with supportive UV spectra.

## Results

### Phylogenetic analyses

**Fig 1:**
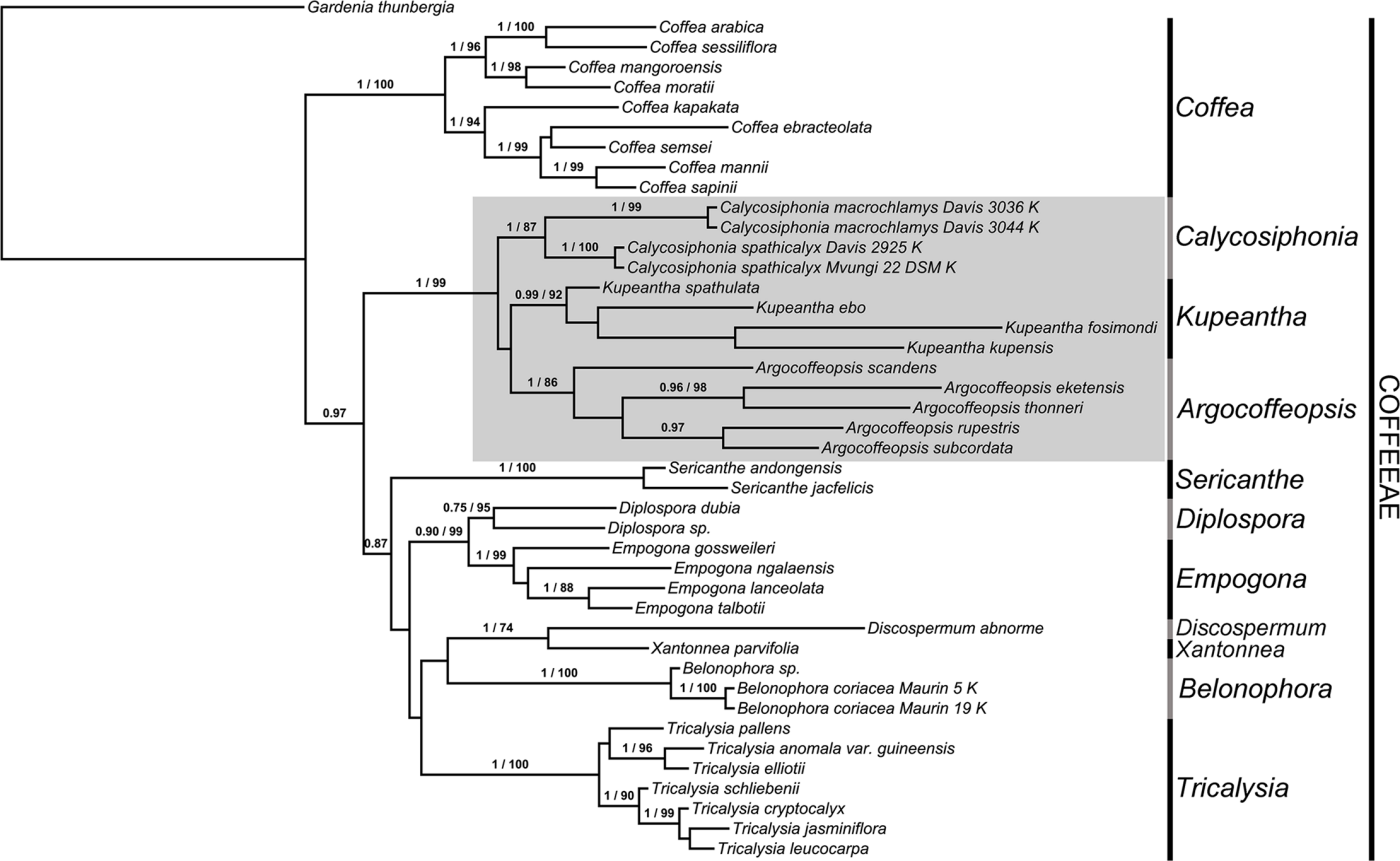
The 50% majority consensus multiple-locus BI tree with the associated PP values and the BS values of the multiple-locus ML tree. Only PP above 0.75 and BS values above 70% are shown. The *Argocoffeopsis* clade is indicated in grey.

The concatenated ML and BI analyses did not generate significantly different topologies, therefore, we present the relationships shown in the 50% majority consensus multiple-locus BI tree, with the associated PP values and the bootstrap values of the multiple-locus ML tree (Fig 1). Only BS values above 70% and posterior probabilities (PP) above 0.75 are shown. The best scoring ML tree is available for comparison in S1 Fig.

Tribe Coffeeae is recovered as monophyletic, with the monophyletic genus *Coffea* strongly supported as sister to the rest of the tribe (PP = 1, BS = 100). The rest of the Coffeeae is represented by two main clades. The first of these, the *Argocoffeopsis* clade (PP = 1, BS = 99) indicated in grey (Fig 1), includes the genus *Argocoffeopsis* (PP = 1, BS = 86), which becomes monophyletic after exclusion of *Argocoffeopsis fosimondi* and *A*. *spathulata*, the monophyletic genus *Calycosiphonia* (PP = 1, BS = 87), and a monophyletic group (PP = 0.99, BS = 92) here named *Kupeantha* comprising *Argocoffeopsis fosimondi* and *A*. *spathulata* in addition to two new species described in this study. The placement of *Calycosiphonia pentamera* in *Kupeantha* was inferred based only on its morphology because DNA could not be extracted.

The other main clade includes the rest of the generic diversity of the tribe. Included in this study are members of *Sericanthe*, *Diplospora*, *Empogona*, *Discospermum*, *Xantonnea*, *Belonophora*, and *Tricalysia*.

### Morphology and palynology

Characters separating *Argocoffeopsis*, *Calycosiphonia* and *Kupeantha gen*. *nov*. are provided in Table 1. Characters separating *Argocoffeopsis fosimondi*, *A*. *spathulata*, *Calycosiphonia pentamera*, and the new species are documented in Table 2. The descriptions in the taxonomic treatment largely follow [48].

**Table 1:** Characters separating *Argocoffeopsis*, *Calycosiphonia* and the new genus *Kupeantha*. Characters diagnostic for *Kupeantha* shown in bold. Number of ovules per locule based on Sonké *et al*. (2007) and Robbrecht (1981).

	*Argocoffeopsis*	*Calycosiphonia*	*Kupeantha*
Habit	Climbing shrubs	Treelets	Small shrubs to treelets
Evergreen or deciduous	Deciduous or Evergreen	Evergreen	Evergreen
Inflorescences	Terminal or axillary & opposite brachyblasts with a series of 2–4 pairs of reduced leaves (homologous with cupular calyculi with foliar lobes) 2.5 cm long	Appearing axillary, fasciculate, terminal on contracted brachyblasts, with leaf pairs reduced to 2–3 pairs cupular calyculi, some of foliar lobes foliose 3.5mm long	Appearing axillary, fasciculate. Terminal on contracted brachyblasts, with leaf pairs reduced to 2–4 cupular calyculi, the **foliar lobes minute, c. 0.2 mm long**
Lateral buds	Axillary, concealed in stipule sheaths	Axillary, concealed in stipule sheaths	**Supra-axillary, emerging from stem 1–4 mm above stipule sheaths, spike-like**
Epidermis of leafy branches (dried specimens)	Pale brown or grey, hairy or (3/8 spp.) glabrous	Black only for a few mm of the most distal internode, otherwise white or pale brown, slightly spongy.	**Black (distal (1**–**)2(**–**4) internodes, proximal internodes white, spongy**
Leaf-blade venation	Tertiary nerves conspicuous. Quaternary nerves usually conspicuous, finely reticulate.	Tertiary nerves conspicuous. Quaternary nerves obscure ± scalariform	**Tertiary & quaternary nerves obscure & inconspicuous** (except *K*. *pentamera* where both conspicuous & finely reticulate). Not scalariform.
Calyx	Tube distinct, c. ¼ length of ovary, with minute lobes	Tube well-developed, about half as long as ovary, lobes absent	**Tube reduced to a minute rim, (shorter than disc), lobes absent**
Anthers	Non-locellate	Locellate	Non-locellate
Terminal connective appendage	Absent or present	Absent	Present
Staminal filament length	(1–)2–3 mm	2–3 mm	**0.2**–**0.5** (–2.2 mm in *K*. *fosimondi*)
Anther attachment	± basifixed	Medifixed	± basifixed
Pollen	(3–)4–5-colporate	3-colporate	3-colporate (observed only in *K*. *kupensis*)
Ovules per locule	1	2 (rarely 1)	1
Leaf blade domatia	Present	Absent or present	Absent

**Table 2:** Significant characters distinguishing *Kupeantha ebo*, *K*. *fosimondi* (partly based on [48]), *K*. *kupensis*, *K*. *pentamera* (partly based on [23]) and *K*. *spathulata* (partly based on [22]).

	*K*. *ebo*	*K*. *fosimondi*	*K*. *kupensis*	*K*. *pentamera*	*K*. *spathulata*
Habit and height (m)	Treelet 5	Shrub (1.5–)2–3	Small tree (2–)3–8	Shrub 0.5–1.5	Shrub 0.5–2.5
Leaf-blade dimensions (cm)	(9–)18–22 x (3–)5.5–7.2	16–21.5 × 4.5–9.5	13–18(–20.5) × (5.7–)6.2–7.3	(13–)14–20 × 4–7	(8.5–)11–17 × 2.5–5.7
Leaf acumen	acuminate to caudate (long cm) (0.9–)1.38–1.6 (–2.3)	Tapering to acute apex	Acumen tapering, slender, acute	Acumen tapering, slender, acute	Spatulate, apex swollen, orbicular
Secondary nerves	6–8, with intramarginal hooped nerves	6–9, intramarginal hooped nerve not apparent	8–10, uniting near the margin forming a looping connecting nerve	(9–)10–13, ascending, straight to curved	7–9, joining to form hooped intramarginal nerve
Petiole length (mm)	(6–)9–13	(9―)11–17	7–17	(7–)9–13	(2–)4–7(–8)
Lower calyculus dimension (mm)	1.3–1.9 x 1.9–2.5 (l x w)	1.6–2 × 2–3.4	c. 2 × 2.8	0.5–1 × 0.5–1	0.5–1 × 1–1.5
Upper calyculus dimension (mm)	1.85–2.3 x 4.2–4.5	2–3 × 3–4	2–3 x 2.5–2.8	1–1.5 × 1–2	0.7–1 × 1.5–2
Corolla tube length (mm)	?	8–10	10–12	10–12	4.5–6(–10)
Corolla lobe length (mm)	?	16–20	12–17	6–7.5	(2.5–)7–9
Anther apex & length (mm)	?	Apiculate, c. 9	Apiculate, 9.2–10.3	Apiculate, c. 2	Subacute, 2.5–3
Fruit shape, size (mm) & apex	Ellipsoid to Globose 1.0–1.6 x 1.1–1.5 cm	Globose, c. 30 × 25, round	Ellipsoid, 22–27 × 14–17, rostrate	Ellipsoid, 17–25 x 13–16.5, round	Obovoid, 12–15 × 5–10, round

**Fig 2:**
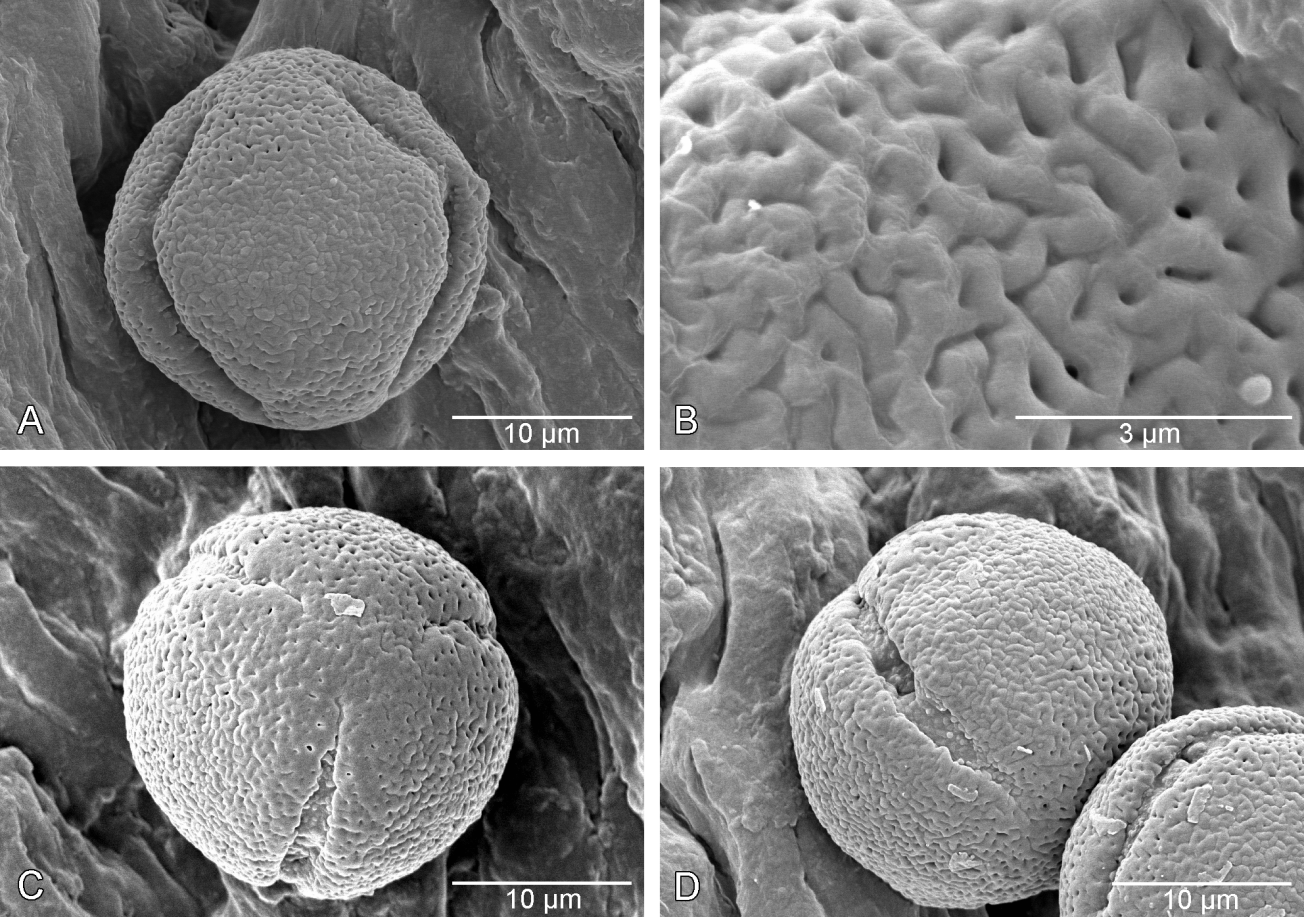
SEM micrographs of pollen of *Kupeantha kupensis*. A. whole grain equatorial view, showing mesocolpium, B. close-up of exine sculpturing in mesocolpial area, C. whole grain polar view, showing apocolpium, D. view of colpus and pore. All from *Cheek 7882* (K).

The pollen grains in *Cheek 7882* (Fig 2) are spheroidal, 19–20 μm in diameter, tricolporate, with an apocolpium of 5–6 μm diameter (Fig 2A). The mesocolpium sculpturing is vermiculate-reticulate (Fig 2B), the projecting elements rounded, about 400 nm wide. Reticulum units are about 800 nm in diameter. The apolcolpium exine sculpturing grades to porate (Fig 2C). The colpal membrane is densely verrucate (Fig 2D) and the pore about 3 μm in diameter.

### Phytochemical analysis

LC–UV–MS/MS seed analysis (Table 3) revealed the detection of a range of hydroxycinnamic acid derivatives, including caffeoyl-, coumaroyl- and feruloyl-quinic acids, in all three extracts, one of *Kupeantha kupensis* (*Cheek* 7882), and two of *Kupeantha ebo* (*Alvarez* 11, *Fenton* 134), which were assigned from their observed [M+H]^+^ ions, with supportive UV spectra and MS/MS interpretation (Table 3). The main hydoxycinnamic acid detected in the positive ionisation mode in the three extracts with *m/z* 339.1070 eluted at the retention time (Rt) of 11.0 min was assigned as 5-*O*-coumaroylquinic acid. Amino acids were also detected in all three extracts and were assigned from their observed [M+H]^+^ ions and supportive MS/MS interpretation as asparagine, phenylalanine and tryptophan, or their isomers (Table 3).

**Table 3:** Compounds assigned from LC-UV-MS/MS analysis of the *Kupeantha kupensis* seed extracts.

Assigned compound (or isomer)	Retention time (min)	Molecular formula	(*m*/*z*)	Ion	*K*. *ebo* (*Fenton 134*) ppm	*K*. *ebo* (*Alvarez 11*) ppm	*K*. *kupensis* (*Cheek 7882*) ppm
Asparagine	1.6	C_4_H_8_N_2_O_3_	133.0607	[M + H]^+^	0.441	2.320	0.742
Phenylalanine	3.0	C_9_H_11_NO_2_	166.0859	[M + H]^+^	2.259	2.439	2.921
Tryptophan	5.0	C_11_H_12_N_2_O_2_	205.0967	[M + H]^+^	1.044	1.434	2.019
3-*O-*Coumaroylquinic acid*	6.9	C_16_H_18_O_8_	339.1068	[M + H]^+^	0.248	1.427	1.781
Caffeoylquinic acid*	7.4	C_16_H_18_O_9_	355.1018	[M + H]^+^	0.004	0.531	1.545
5-*O*-Coumaroylquinic acid*	11.0	C_16_H_18_O_8_	339.1070	[M + H]^+^	1.339	2.223	2.577
Feruloylquinic acid*	12.5	C_17_H_20_O_9_	369.1173	[M + H]^+^	0.403	0.240	1.974
4-*O*-Coumaroylquinic acid*	14.0	C_16_H_18_O_8_	339.1071	[M + H]^+^	0.955	0.601	0.018
Villanovane I^a^	15.5	C_26_H_40_O_9_	497.2737	[M + H]^+^	0.682	0.059	1.728
Tricalysiolide A^b^	19.6	C_20_H_28_O_4_	333.2059	[M + H]^+^	Nd	Nd	0.408
Tricalysioside E^b^	21.6	C_32_H_48_O_14_	657.3110	[M + H]^+^	Nd	Nd	1.084
Tricalysiolide B^b^ or G^b^ or tricalysin D^c^	23.1	C_20_H_28_O_5_	349.2008	[M + H]^+^	Nd	Nd	0.488
Tricalysioside B^b^ or C^b^	24.4	C_26_H_38_O_9_	495.2575	[M + H]^+^	0.103	1.190	2.664
Tricalysioside P^b^	34.8	C_26_H_42_O_9_	516.3161	[M + H]^+^	0.946	0.830	0.345
Feruloyl hexoside	36.4	C_16_H_20_O_9_	357.1178	[M + H]^+^	Nd	Nd	0.500

All compounds assigned by comparison of accurate mass data (based on ppm = parts per million), and by interpretation of available MS/MS and/or UV spectra.

Nd: Not detected / below level of detection.

*Reported to occur in coffee (*Coffea* species) and numerous other plant species [27, 49, 50].

^a^Reported to occur in puer coffee beans (*Coffea arabica* ‘Yunnan’) [27].

^b^Reported to occur in *Tricalysia dubia* (Lindl.) Ohwi [26], accepted name *Diplospora dubia* (Lindl.) Masam.

^c^Reported to occur in *Tricalysia fruticosa* (Hemsl.) K.Schum. ex E.Pritz. [27], accepted name *Diplospora fruticosa* Hemsl.

In the present study, a compound eluting at Rt 15.5 min with *m/z* 497.2737 was detected in both new species and was assigned with the molecular formula C_26_H_40_O_9_, determined from the [M+H]^+^ ion, which is that of the diterpenoid glucoside, villanovane I. Also, detected in all of the extracts analysed were compounds eluting at Rt 24.4 min and 38.8 min, with *m/z* 495.2575 and 516.3161, respectively. These compounds were assigned with the molecular formulae from their observed [M+H]^+^ ions as C_26_H_38_O_9_, which is that of tricalysioside B or C, and as C_26_H_42_O_9_, which is that of tricalysioside P. Other compounds assigned as *ent*-kaurane diterpenoids or their glycosides were only detected in the seed extract of *Cheek 7882*. These included a compound eluting at Rt 19.6 min with *m/z* 333.2059 that was assigned with the molecular formula C_20_H_28_O_4_, determined from the observed [M+H]^+^ ion, which is the molecular formula of tricalysiolide A. Also detected in the seed extract of *Cheek 7882* were compounds eluting at 21.6 min and 23.1 min, with *m/z* 657.3110 and 349.2008, respectively. These compounds were assigned with the molecular formulae C_32_H_48_O_14_ and C_20_H_28_O_5_ from their observed [M+H]^+^ ions, which are the molecular formulae of tricalysioside E, and of tricalysiolide B or G (or tricalysin D), respectively.

## Discussion

### Relationships in tribe Coffeeae

The relationships recovered in tribe Coffeeae (Fig 1) largely match those obtained in previous studies [21, 34], with *Coffea* sister to the rest of the tribe, but with little support for the relationships among most genera. [34] removed the species of *Empogona* from *Tricalysia* since they are more closely related to the genus *Diplospora*. This relationship is confirmed here (Fig 1). As in [21], we find a possible sister relationship between *Discospermum* and *Xantonnea*, although only a single taxon of each was sampled and therefore, this relationship needs further investigation. According to our results (Fig 1), species previously included in *Argocoffeopsis* and *Calycosiphonia*, in addition to two new taxa, form a well-supported monophyletic group. This clade is here indicated as the *Argocoffeopsis* clade, which is the generic name with priority.

### Generic delimitation of *Argocoffeopsis* and *Calycosiphonia*

Within the *Argocoffeopsis* clade, three well-supported subclades are recovered: (1) *Calycosiphonia* (excluding *C*. *pentamera* due to unavailability of DNA, but transferred to *Kupeantha* based on morphology), (2) *Argocoffeopsis* (excluding *A*. *fosimondi* and *A*. *spathulata*), and (3) *Kupeantha*, a clade including the above excluded species, in addition to the newly described *K*. *kupensis* and *K*. *ebo* (Fig 1). These three subclades are supported by numerous morphological characters (Table 1).

### Generic delimitation of *Kupeantha* and its species

The morphological affinities of the new species, *K*. *kupensis* and *K*. *ebo*, shown in Table 2, appear to lie with *Calycosiphonia pentamera*, *Argocoffeopsis fosimondi* and *A*. *spathulata*. In all five species, distinctive supra-axillary buds are developed, and the distal internodes of the stem dry dull black, becoming abruptly white and spongy in one of the more proximal internodes. The lobes of the calyculi are minute, and never foliose, except in deviant cases [19]. The calyx tube is reduced to a minute rim shorter than the disc, and lobes are absent. These characters set this group apart from *Argocoffeopsis* and *Calycosiphonia*. Although these five species, now included in the new genus *Kupeantha*, resemble *Calycosiphonia* in being evergreen small trees or shrubs, with glabrous leaves and axillary, calyculate, 1-flowered inflorescences, they lack the locellate anthers and 7–8-merous flowers found in that genus. Instead, flowers are (5–)6-merous, with non-locellate anthers. Superficially, they seem unrelated to *Argocoffeopsis* since they appear not to bear flowers on short leafy spur shoots.

*Kupeantha kupensis* differs from all other species of *Kupeantha* in having ellipsoid-rostrate fruits (the other species lack rostrate fruits). The long (12–17 mm) corolla lobes are only exceeded by those of *Kupeantha fosimondi* (16–20 mm) and these two species share characters not seen in *Kupeantha pentamera* and *Kupeantha spathulata*, such as large anthers 9–10 mm long (instead of anthers c. 2 mm long), and a submontane habitat (instead of a lowland habitat). The affinities of *Kupeantha ebo* are more difficult to ascribe since flowers were lacking. In shape and size of leaves and fruits it resembles most closely *K*. *pentamera*, but differs by lacking the finely reticulate nervation of that species (the quaternary nerves are inconspicuous), and by having 6–9 secondary nerves (not 10–13) on each side of the midrib. Keys to the genera, species descriptions, and nomenclatural changes are provided in the taxonomic treatment below.

### Phytochemical analysis

Hydroxycinnamic acid derivatives are known constituents of the seeds of *Coffea* species, which are used to prepare the beverage coffee [49, 50], and were also detected in all of the extracts of *Kupeantha kupensis* and *Kupeantha ebo* analysed in the present study. However, whilst the purine alkaloid caffeine is widely documented to occur in *Coffea* seeds, it was not detected in any of the seeds analysed in this study, nor in other previously studied genera in the Coffeeae [32].

A compound assigned as villanovane I (or isomer) was detected in both *Kupeantha ebo* and *K*. *kupensis*, and is a diterpenoid glucoside previously reported to occur in *Coffea* species [27]. Additionally, compounds assigned as tricalysioside B or C, and as tricalysioside P (or their isomers) were detected. These *ent*-kaurane glycosides have been reported to occur in species previously placed in the genus *Tricalysia* and now included in *Diplospora* [27]. Other compounds assigned as *ent*-kaurane diterpenoids or their glycosides were only detected in *Kupeantha kupensis*. These included compounds assigned as tricalysiolide A, tricalysiolide B or G (or tricalysin D), and tricalysioside E (or their isomers). These *ent*-kaurane compounds have also been reported to occur in species now placed in *Diplospora* [27], as indicated in Table 3. In summary, the detection of hydroxycinnamic acid derivatives, amino acids and *ent*-kaurane diterpenoids, or their isomers, which also occur in *Diplospora* species, are reported here for the first time in seeds of species in the *Argocoffeopsis* clade.

## Taxonomic treatment

A key to the genera in the *Argocoffeopsis* clade is provided in below.

### Key to the genera of the *Argocoffeopsis* clade

1. Climbing shrubs (usually), stems and leaves hairy (5 of the 8 species), flowers terminal, single, on short lateral stems (brachyblasts), with 2–3 pairs of more or less slightly reduced leaves; calyx lobes present...................................................*Argocoffeopsis*

1. Treelets or sparsely branched shrubs >0.5 m tall, glabrous, flowers 1-several in axils, each subtended by 2–3 calyculi (reduced brachyblasts); calyx lobes absent...................................................2

2. Tertiary nerves ± scalariform in leaf-blades; white-pale brown epidermis (dried specimens) developing within mm of the stem apex; axillary buds concealed within stipule sheaths; anthers locellate, lacking apical connective appendage, filaments 2–3mm, calyx tube well-developed, at least 1mm long, accrescent and conspicuous in fruit...................................................*Calycosiphonia*

2. Tertiary nerves absent or inconspicuous, or (*K*. *pentamera*) present with quaternary nerves forming reticulate, not scalariform nervation; white-pale, corky epidermis (dried specimens) developing (1–)2 (–4) internodes below the stem apex; buds supra-axillary, inserted on stem 1 or more mm above the stipule sheath; anthers entire, not locellate, with a terminal connective appendage; filaments 0.2–0.3 mm long, calyx tube c. 0.1 mm long or less, barely detectable, inconspicuous in fruit...................................................*Kupeantha*

***Kupeantha*** Cheek, **gen. nov.** [urn:lsid:ipni.org:names:60476503–2] *Type*: *Kupeantha kupensis* Cheek & Sonké

Differs from *Calycosiphonia* Robbr. and *Argocoffeopsis* Lebrun in the conspicuous, spike-like, supra-axillary buds, which are raised 1–4 mm above the stipule sheaths (not axillary, concealed within the stipule sheaths); epidermis of leafy stems drying black in the distal (1–)2(–4) internodes, becoming white and spongy in proximal internodes (not pale brown or grey, or, if black, only for a few mm from the most distal internode); calyx tube a minute indistinct rim above the hypanthium, shorter than the disc; calyx lobes absent (not with a distinct tube longer than disc with minute lobes or with a well-developed tube, half as long or more than ovary); the calyculi with foliar lobes minute, c. 0.2 mm long (not with foliar lobes 3.5 mm or more long, or with calyculi not formed).

Shrubs or small trees, glabrous, hermaphrodite, evergreen. Stems drying black in the distalmost (1–)2(–4) internodes, terete, changing abruptly proximally to a thicker, spongy, bright white epidermis, at length becoming, in older, non-leafy stems, pale brown and flaking-fibrous; lenticels absent. Stem apex with translucent gum from stipule colleters, as in *Coffea*; internodes even in length; axillary buds spike-like 2–4 mm long, supra-axillary, raised above the stipule sheath by several millimetres. Leaves opposite, equal in size and shape at nodes, and distichous. Leaf-blades elliptic, oblong-elliptic, rarely slightly obovate (*K*. *pentamera*), apex acuminate, rarely acute, base broadly acute, usually slightly asymmetric, margins entire, often slightly revolute, secondary nerves 7–13 on each side of the midrib, domatia absent, nervation usually brochidodromous, tertiary and quaternary nerves usually obscure (except in *K*. *pentamera*, where together they form a fine reticulum). Petiole plano-convex in transverse section, convex below, and flat above, not articulated with stem. Stipules sheathing with a triangular limb, often extending into an awn-like projection, inner surface with scattered standard colleters and simple hairs. Inflorescences above the axils of leaves, opposite, sometimes in successive nodes, 1(–3) per axil, or (*K*. *kupensis*) 2–4 per axil, each calyculate, 1-flowered, terminal. Calyculi 2–3, or (*K*. *kupensis*) 3(–4), completely concealing the inflorescence axis and the base of the ovary-hypanthium, 4-lobed (2 foliar lobes and 2 stipular lobes) or (proximal calyculi) lacking lobes, glabrous outside, but with colleters, and sometimes hairs, inside, sometimes on the margin; calyculi cup-like or shortly cylindrical, the most distal largest; foliar lobes usually ovate, non-foliose; stipular lobes, if developed, ± triangular, about as long as foliar lobes. Peduncles absent; rachis completely concealed by calyculi, <1(–2) mm long. Flowers 5–6-merous, homostylous. Calyx (hypanthium) entirely contained within the upper calyculus at anthesis, rarely with up to 1 mm of the apex exserted, shortly cylindric, glabrous; calyx limb truncate, glabrous, lacking colleters inside, 0.1–0.2 mm long, shorter than disc, barely detectable. Corolla glabrous, white; corolla lobes contorted to the left in bud; corolla tube cylindrical, widening slightly at the apex, about as long as the lobes; corolla lobes oblong-elliptic, apices asymmetrically shortly acuminate. Anthers introrse, fixed at apex of the corolla tube at the base of the lobes, completely exserted, submedifixed; filaments short (except in *K*. *fosimondi* to 2.5 mm), inserted c. 1 mm from the base of the anther; anther sacs oblong, not locellate, base shortly bifid, apex with connective minutely apiculate. Pollen globose, tricolporate, surface vermiculate-reticulate (Fig 2). Disc subcylindrical, glabrous. Ovary bilocular, style filiform, glabrous, fleshy, stigma arms 2, separated or partly appressed together, exserted. Fruits ripening orange, yellow, globose, obovoid or ellipsoid, 12–30 mm long, sometimes shortly stipitate or rostrate, disc and calyx limb not markedly accrescent; outer surface matt, subrugulose, mesocarp leathery (becoming fleshy?); endocarp distinct, brown, glossy, vascularized, translucent, membranous to thinly papery. Seeds 1–2, plano-convex and elliptic-orbicular in outline 8–13 mm long (where 2 seeds per fruit) lacking grooves, but sometimes with the radicle emerging slightly at one end. Seed coat not detected. Endosperm waxy, translucent orange-brown, forming 98% of the volume of the seed. Embryo visible within the intact seed in transmitted light, straight, linear, running along the axis of the seed, radicle pointing downwards (inferior).

Etymology: *Kupeantha* means “flower of Kupe”.

Five species in lowland and submontane evergreen forest in Cameroon, one species extending to Rio Muni (Equatorial Guinea). A key to the species of *Kupeantha* is provided in below.

### Key to the species of *Kupeantha*

1. Fruit obovoid; acumen spathulate; petiole <1cm long...................................................*K*. *spathulata*

1. Fruit globose or ellipsoid; acumen with apex acute; petiole >1cm long...................................................2

2. 0.5–1m tall; quaternary nerves conspicuous, forming fine reticulation on lower surface of leaf-blade; secondary nerves 10–13 on each side of midrib; leaves drying black...................................................*K*. *pentamera*

2. 2–5m tall; quaternary nerves absent or inconspicuous, fine reticulation absent; secondary nerves <10 on each side of midrib; leaves drying green...................................................3

3. Fruits ellipsoid, ripening black, with a short rostrum SW Region, Mt Kupe...................................................*K*. *kupensis*

3. Fruit globose, ripening orange-red, stipe and rostrum absent or inconspicuous. SW Region, Lebialem or Littoral Region, Ebo...................................................4

4. Fruit 25–30 mm diameter. Lebialem Highlands of S.W. Region; 1300–1400 m elev.....*K*. *fosimondi*

4. Fruit 10–15 mm diameter. Ebo Highlands of Littoral Region; 770–830 m elev....................................................*K*. *ebo*

**Fig 3:**
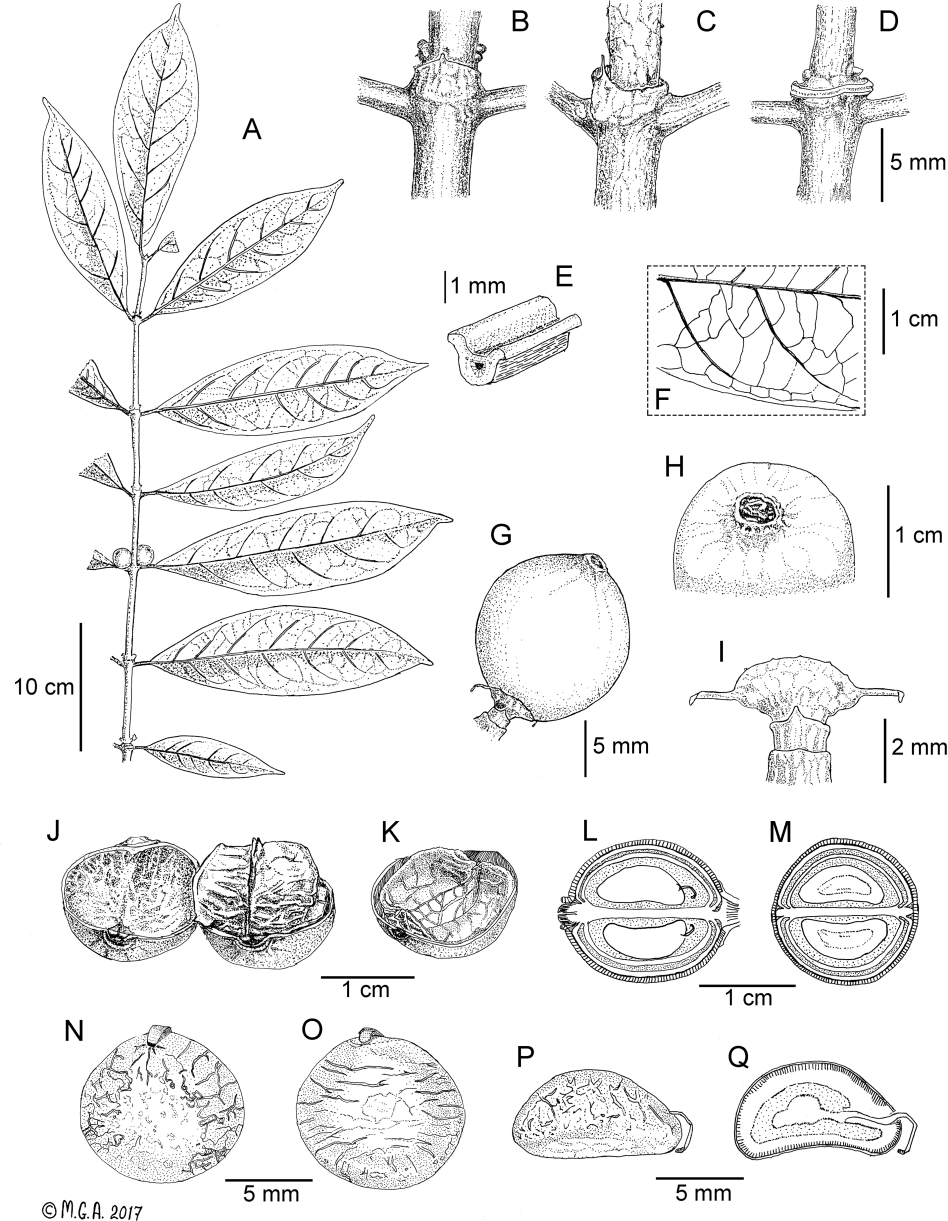
*Kupeantha ebo*. A. fruiting branch, B detail of stipule and supra-axillary buds, C, D. detail of stipule and stipule scar at an older nodes, E. transverse section of petiole, F. nervation, abaxial surface of leaf, G. fruit, H. fruit apex showing disc, I. Calyculi, J. opened fruit showing the two endocarps with sagittal crest surrounding the seeds, K. half fruit showing the vascularised membrane (endocarp) separating the two seeds, L. diagram of median longitudinal section of a fruit, showing the exocarp (shaded area), mesocarp (white areas), endocarp, cavities (dotted areas) and two seeds with emergent embryos, M. diagram of a median cross section of a fruit, N. dorsal view of seed, O. ventral view of seed, P. lateral view of seed, Q. median longitudinal section of seed showing the emergent embryo. A–C, E–G, I from *Alvarez 11* (K); D, H, J–Q from *Fenton 134* (K). Drawing by Maria G. Alvarez-Aguirre.

**1. *Kupeantha ebo*** M. Alvarez & Cheek, **sp. nov.** [urn:lsid:ipni.org:names:60476504–2] (Fig 3).

Type: CAMEROON, **Littoral Region:** Ebo Proposed National Park, near the Njuma River, near Njuma Camp, 770 m elev., 4°21'26” N, 10°15'01” E, fr., 3.x.2015, *M*. *Alvarez 11* (holotype: K; isotype: YA).

Diagnosis: Differs from *K*. *pentamera* by being a small tree or shrub 2–5m high, not a shrublet 0.5–1m tall, and having fruits 10–16 mm long (not 17–25mm long); secondary nerves 6–8 (not 10–13), on each side of the midrib; tertiary nerves not conspicuous, versus conspicuous and forming a fine reticulum.

Description: Small tree or shrub, 2–5 m high. Stem dark, greyish brown to lighter reddish brown. Stem terete, glabrous, smooth to flaky and peeling off on older stems, 4–5 mm in diameter. Stipules connate, subcoriaceous, glabrous outside, puberulous inside, with gum exudate, rectangular to trapezoid, 1.5–2.8 mm long × 2.6–4.5(–6) mm wide, apex mucronate to narrowly triangular, crustaceous, 0.8–2 mm long. Leaves opposite, equal; petioles glabrous, drying darker and shinier than stems, (6–)9–13 mm long; leaf–blades drying dark green above and light green below, narrowly elliptic to oblong, glabrous above and below, cartaceous, (9–)18–22 × (3–)5.5–7.2 cm; apex tapering, acuminate to caudate (9–)13–16(–23) mm long, base attenuate; length/width ratio 3:1 to 4:1. Midrib sub-prominent above and prominent below; secondary nerves impressed above and prominent below, 6–8 on each side, ascending and curving to make a looping intramarginal nerve (brochidrodomous); midrib and secondary ribs drying dark brown below. Tertiary nerves conspicuous, reticulate above and weakly percurrent below. Quaternary nerves inconspicuous. Domatia absent, bacterial infections like ulcers of irregular form may appear on blades or on veins. Inflorescences on leafy branches. Flowers not seen. Inflorescences in pairs at each node, 1 per axil, supra-axillary, situated 1.5–1.8 mm above the base of petioles. Calyculi 2 to 3, sessile, cupuliform glabrous. The first calyculus (basal), may be corky, without apparent lobes, sometimes it forms part of the stem, 1.3–1.9 × 1.9–2.5 mm, the second one smooth, with 2 broadly triangular stipular lobes 2.1–2.3 × 1.8–3 mm; the third distal one cupuliform at the base, smooth, with a lenticular structure at the top, 1.8–2.3 × 4.2–4.5 mm, sinuate margin with 2 very fragile subulate lobes, c. 1.5 x 0.2 mm. Fruits berry like, ellipsoid when immature to globose when mature, 1.0–1.6 × 1.1–1.5 cm. Orange to red at maturity, glabrous, exocarp drying hard, leathery, dark brown; disc ± circular, 2.8–4 mm in diameter; calyx limbs absent. Disc exserted in young fruits, becomes ± flat in older fruits. Endocarp membrane very vascularised, separating the seeds. Seeds: 1–2, apparently without seed coat, surface ± smooth, endosperm entire cream. Where 2 seeds, the seed is plano–convex, broadly ovate in outline, c. 1 × 0.8 × 0.4 cm. Where 1 seed, ellipsoid with irregular surface, circular in cross section, c. 1.1 × 0.7 cm. Micropyle visible on seed embryo can be seen germinating prematurely in some seeds.

Distribution and habitat: *Kupeantha ebo* is endemic to the Ebo Proposed National Park in the Littoral Region in Cameroon. The species is found in submontane, evergreen forest, on well-drained sandy to loamy and rocky soil from 770 to 832 m in elevation. The diversity is represented by large trees of *Aphanocalyx microphyllus* (Harms) Wieringa, *Berlinia bracteosa* Benth., *Cynometra hankei* Harms, *Distemonanthus benthamianus* Baill., *Hymenostegia viridiflora* Mackinder & Wieringa, *Plagiosiphon longitubus* (Harms) J.Léonard, *Pterocarpus soyauxii* Taub. (all Fabaceae); *Terminalia superba* Engl. & Diels (Combretaceae) and *Vitex lokundjensis* W.Piep. (Lamiaceae).

Phenology: Flowering unknown; fruiting in October.

Conservation status: *Kupeantha ebo* is restricted to the Littoral Region in Cameroon and it is known from only two sites in Ebo Proposed National Park [51,52]. The forest, where this new species was found, was a logging and farming area and it currently seems to be slowly regenerating, since these activities stopped some years ago, although there are still gaps of secondary vegetation among the primary forest. Considering the Ebo Forest does not have a protected status, the survival of this species will depend on the collaboration between locals and the scientific community. There are threats from oil palm plantations and iron ore mining, in addition to the resumption of slash and burn small-holder agriculture. Given that several botanists have spent cumulatively many months over several years collecting specimens at Ebo, and that only two specimens of this species have resulted, it is clear that *K*. *ebo* is genuinely infrequent and rare. Accordingly, *K*. *ebo* is here assessed as Critically Endangered CR B2ab(iii) [45].

Additional specimens examined: CAMEROON, **Littoral Region:** Ebo Proposed National Park, Yabassi, Bekongo trail. 832 m elev. 4°20'45 N, 10°24'40 E. fr. 08 Oct. 2007. *E*. *Fenton 134* (K).

**2. *Kupeantha fosimondi*** (Tchiengué & Cheek) Cheek, **comb. nov.** [urn:lsid:ipni.org:names:60476505–2] ≡ *Argocoffeopsis fosimondi* Tchiengué & Cheek, Pl. Lebialem Highlands: 54 (2010).

Type: CAMEROON, W Bamboutos Mts, Ntoo forest at Fosimondi Forest, 1350 m elev., 24.ii.2006, *B*. *Tchiengué 2597* (holotype: sheet 1/2 K000518627, sheet 2/2 K000265593; isotypes: K000265585, WAG0247275).

**Fig 4:**
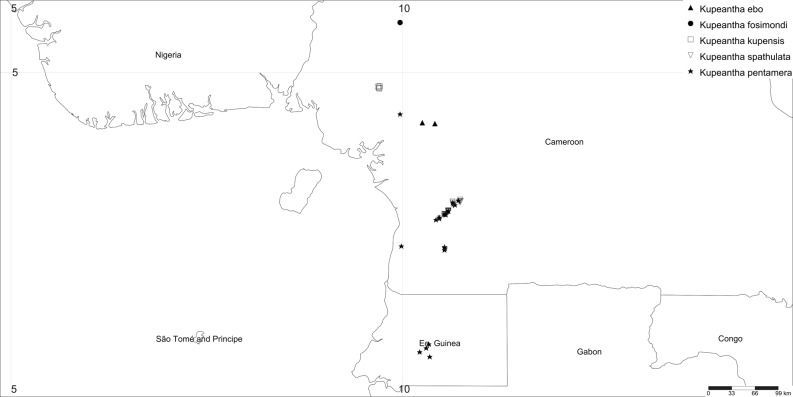
Known area of distribution of the five species of *Kupeantha*.

Distribution: Cameroon, only known from the Lebialem Highlands (Fig 4).

**Fig 5:**
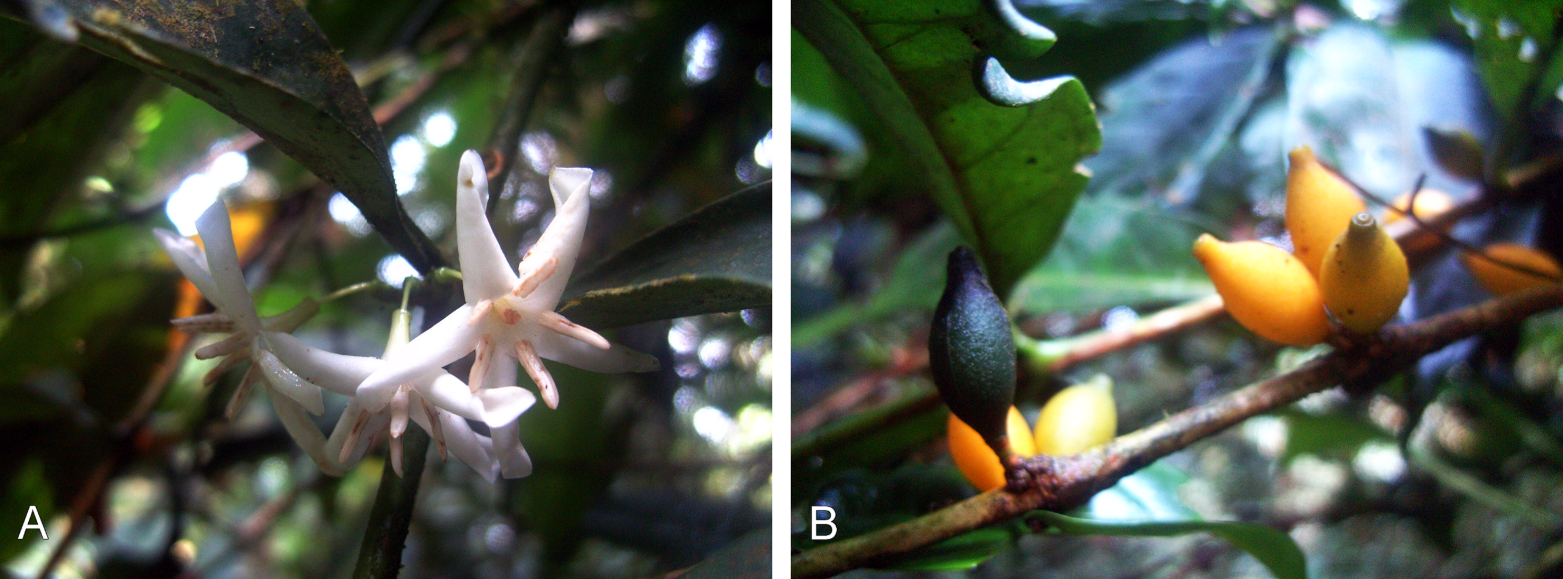
Field photographs of *Kupeantha kupensis*. A. detail of flowers, B. detail of immature and mature fruits. Photos taken from *Cheek 7882* at Mt Kupe, Cameroon, by M. Cheek.

**Fig 6:**
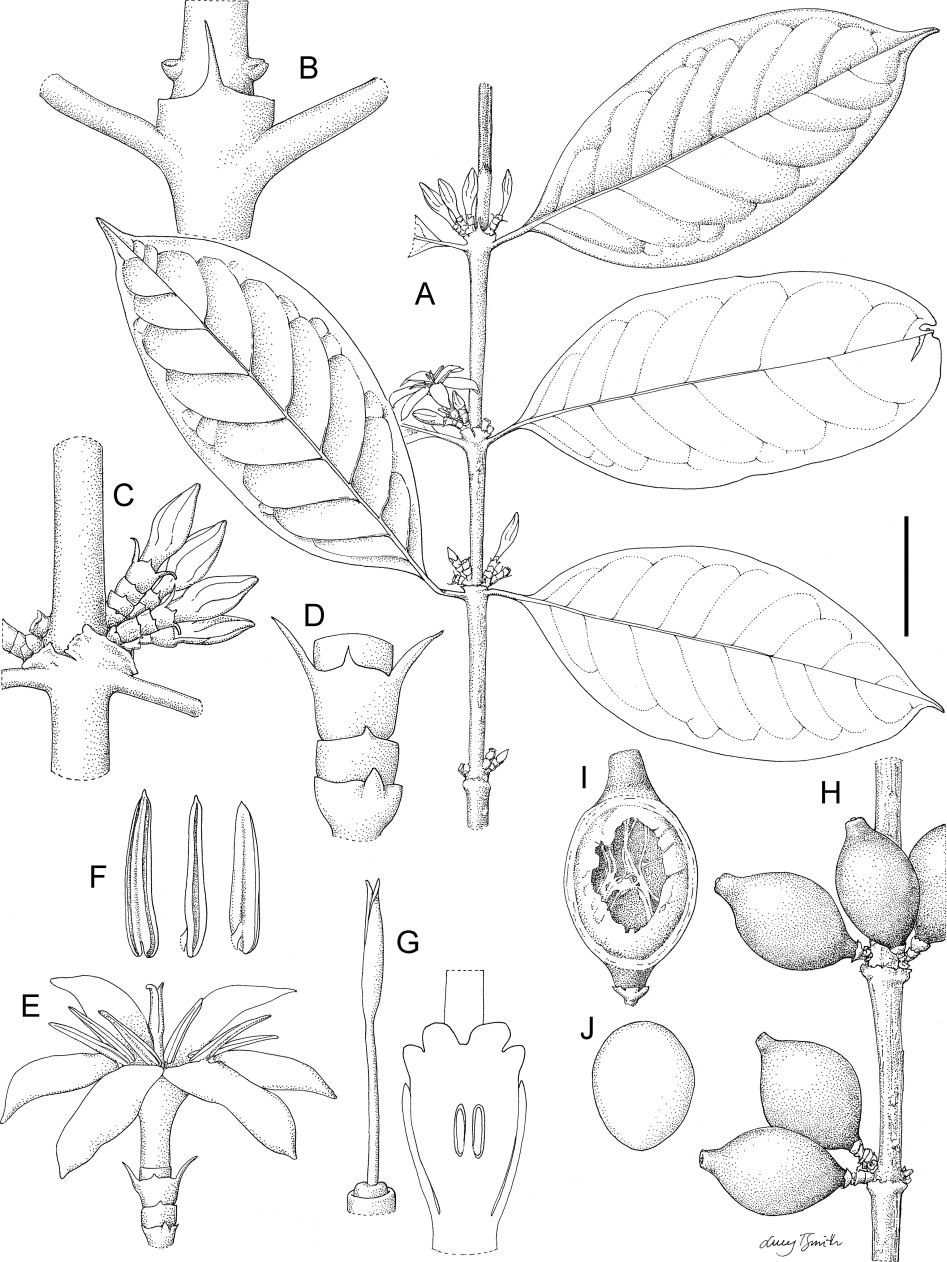
*Kupeantha kupensis*. A. habit, flowering branch, B. detail of stipule and node and supra-axillary buds, C. node with inflorescences, flowers in bud, D. calyculi, E. flower and calyculi, F. stamens, different views, G. gynoecium, left entire, showing disc and calyx, right, longitudinal section, H. fruits, I. fruit with a longitudinal section, showing empty locule (seed removed), J. seed, ventral surface. Scale bar: A = 4 cm; B, F, G = 7 mm; C, E, J, K = 1 cm; D = 4 mm; H = 2.5 mm; I = 2 mm. Drawn by Lucy T. Smith from *Cheek 7882* (K).

**3. *Kupeantha kupensis*** Cheek & Sonké, **sp. nov.** [urn:lsid:ipni.org:names:60476506–2] (Figs 5 and 6).

Type: CAMEROON, Cameroon Highlands, Mt Kupe, above Kupe Village, 1550 m elev., 4°48’N, 9°42’E, fl. fr., 17.xi.1995, *Cheek 7882* (holotype: K; isotypes: BR, MO, P, SCA, US, WAG, YA).

Previously mentioned in literature as: *Calycosiphonia* sp. A [6: 369] and *Argocoffeopsis kupensis* Cheek & Sonké ined. [5: 260 & map 560E].

Diagnosis: differs from *K*. *spathulata* and *K*. *fosimondi* by having ellipsoid and rostrate fruits (not globose, apex rounded), corolla tube 11–12 mm long (not 4–5 mm long), lobes 12–17 mm long (not 7–9 mm as in *K*. *spathulata*, nor 16–20 mm as in *K*. *fosimondi*).

Description: Shrub or small tree, (2–)3–8 m tall, glabrous; main axis brown. Stem terete, 2.5–4 mm diam., drying matt black in first and second internodes, in the third to fourth internodes the epidermis becoming white, spongy, subglossy and exfoliating as in a *Chazaliella* E.M.A.Petit & Verdc.; internodes (2.8–)5.2–6.8 cm long, apical bud with gum covering; buds supra-axillary, covered in gum, inserted 3–4.5 mm above the petiole bases. Petiole 0.7–1.7 cm long, drying black. Leaf blades opposite, equal, elliptic-oblong, rarely ovate-lanceolate, 13–18(–20.5) × (5.7–)6.2–7.3 cm, acumen tapering, slender, acute, 1 cm long, 3–4 mm wide at base, base obtuse to acute, slightly to conspicuously asymmetric, midrib impressed above, lateral nerves 8–10 on each side of the midrib, arising at c. 50 degrees from the midrib, curving upwards and uniting near the margin to form a weak, looping, connecting nerve, tertiary and quaternary nerves sparse, reticulate, not conspicuous, domatia absent; margin entire; drying very dark green above, pale green below, with midrib nearly black; petiole flattened above, concave below, 0.7–1.7 cm long, drying black. Stipule with basal cylindrical sheath 2–4 × 4–6 mm; truncate, distal limb absent to narrowly triangular or awn-like 1–4 mm long, with central ridge absent. Inflorescences on leafy branches near nodes (2–)3–4 internodes from apex, subtended by leaves, inserted 2–4 mm above the leaf axil; fasciculate, inflorescences 1-flowered, 2–4 per axil at both axils of node; 2–4 axils fertile per stem. Calyculi 3(–4), the uppermost slightly larger, each calyculus cylindrical-campanulate, more or less 4-lobed (the uppermost: 2 foliar lobes and 2 stipular lobes) or 2-lobed (the lower calyculi, with two foliar lobes only), ± sessile, glabrous, colleters not seen; basal (1^st^) calyculus c. 2 × 2.8 mm, foliar lobes reduced to mucra, c. 0.7 mm long, stipular lobes absent or minute; middle (2^nd^) calyculus resembling the basal calyculus; upper (3^rd^) calyculus 2–3 × 2.5–2.8 mm, foliar lobes narrowly triangular or ligulate, 2.2 × 0.5 mm long, stipular lobes 0.75 mm long. Flowers hermaphrodite, homostylous (5– or) 6–merous, sessile. Calyx (hypanthium) entirely contained within the upper calyculus at anthesis, rarely with up to 1 mm of the apex exserted, c. 3.5 × 2.3 mm, glabrous; calyx limb truncate, glabrous, lacking colleters inside, 0.1–0.2 mm long, barely detectable. Corolla glabrous, white; corolla lobes contorted to the left in bud; corolla tube cylindrical, widening slightly in the apical 2 mm, (9–)10–12 mm long; corolla lobes oblong-elliptic, 12–17 × 3–4 mm, apices asymmetrically shortly acuminate. Anthers fixed at apex of the corolla tube at the base of the lobes, completely exserted, submedifixed; filaments c.1 mm long, inserted c.1 mm from the base of the anther; anther sacs 9–10 mm long, base shortly bifid, apex with connective apiculate, 0.2–0.3 mm long. Disc subcylindrical, c. 0.4 × 1.8–2 mm, glabrous. Ovary bilocular, not dissected; style filiform, 9.8–1.15 cm × 0.5 mm, glabrous, fleshy, stigma arms 2, appressed together, 4–8 mm, only the apical 1–2 mm separated, exserted. Fruits black, at length ripening orange, ellipsoid, 22–27 × 14–17 mm including a 2–3 mm broad, 3–5 mm long rostrum with apex truncate, disc and calyx limb present but not markedly accrescent; outer surface matt, subrugulose, mesocarp leathery (becoming fleshy?), 0.7–1 mm thick when dried; endocarp distinct, brown, glossy, vascularized, translucent, membranous to thinly papery. Seeds 1–2, plano-convex and elliptic-orbicular in outline 10–13 × 8 × 2–3 mm (where 2 seeds per fruit) or c. 9 × 5 × 6 mm (where 1 seed per fruit), lacking grooves, but sometimes with the radicle emerging slightly at one end. Seed coat not detected. Endosperm waxy, translucent orange-brown, forming 98% of the volume of the seed. Embryo visible within the intact seed in transmitted light, straight, linear, running along the axis of the seed, c. 4 mm long.

Distribution and habitat: *Kupeantha kupensis* is restricted to the SW Region in Cameroon (Fig 4). This new species is known only from Mt Kupe. The area from which *K*. *kupensis* is known supports a submontane (cloud) forest with *Santiria trimera* (Oliv.) Aubrév. (Burseraceae), *Cola verticillata* (Thonn.) Stapf ex A.Chev. (Sterculiaceae), *Garcinia smeathmannii* (Planch. & Triana) N.Robson ex Spirl. (Clusiaceae), *Zenkerella citrina* Taub. (Fabaceae-Detarioideae), *Chassalia laikomensis* Cheek, and *Pauridiantha paucinervis* (Hiern) Bremek. (both Rubiaceae), *Carapa* Aubl. spp. (Meliaceae). This evergreen forest has a closed-canopy with many epiphytes and a rich herb layer, classified as Biafran evergreen forests [53], rich in Fabaceae-Caesalpinoideae. The Kupe area is now well known as an area of high endemism in Cameroon [6]. Elevation 1100–1600(–2000?) m.

Phenology: Flowering in February, November; fruiting in January, June, July, October and November.

Etymology: Named for Mt Kupe, the only known location for the species. The survival of this species is in the hands of the Bakossi people and others who live around the mountain.

Conservation status: This assessment maintains that of Cheek [5: 260]. *Kupeantha kupensis* is only known from two sites at a single location. Many thousands of specimens have been collected at Mt Kupe [6] so the area cannot be considered undercollected. Although the Mt Kupe Integrated Ecological reserve has been created in the last 10 years, both sites for *Kupeantha kupensis* fall outside its boundary. One site is under threat of forest clearance (the type collection was made where a coffee plantation had been created inside the forest). *Kupeantha kupensis* is here assessed as Critically Endangered CR B2ab(iii) [45]. B2 indicates that the total area of occupancy is less than 10 km^2^ (in fact the area of occupancy is 8 km^2^ using the IUCN preferred 4 km^2^ cells for each of the two sites known at this location), while b(iii) indicates “continuing decline inferred in area, extent and quality of habitat”. Further exploration is needed to find more locations for this tree, if they exist, however, the western slopes of Mt Kupe have been relatively intensively surveyed for plants. We hope that the people of Mt Kupe will seek to protect this beautiful species from extinction.

Note: *Kupeantha kupensis* is the latest of many narrowly endemic species recently described from Mt Kupe. Other examples are *Diospyros kupensis* Gosline [54], *Memecylon kupeanum* R.D.Stone, Ghogue & Cheek [55], *Dracaena kupensis* Mwachala, Cheek, Eb.Fisch. & Muasya [56] and *Psychotria ngollengollei* Cheek [57].

Additional specimens examined: CAMEROON, Cameroon Highlands, above Nyasoso, Max’s trail, 5 Feb 1984 (fl.), *Thomas* 3087 (K, MO n.v.); ibid., 9 Jul 1992 (young fr.), *Sunderland* 1539 (K, SCA n.v., YA n.v.); ibid., 29 Jan 1996 (fr.), *Etuge* 1664 (K, SCA n.v., WAG n.v., YA n.v.); ibid., 24 Jun 1996 (fr.), *Etuge* 2381 (YA); ibid., 24 Jun 1996 (fr.), *Etuge* 2401 (K, SCA n.v., YA n.v.); ibid., (4°49'19.5'' N 9°42'16.1'' E) 21 Oct 2009 (fr), *Sonké & Taedoumg* 5379 (BR, BRLU, K, MO, WAG, YA); ibid., (4°49'20.00'' N 9°42'16.5'' E), 24 Apr 2009, Dessein & al. 2728 (BR, P, WAG, YA).

**4. *Kupeantha pentamera*** (Sonké & Robbr.) Cheek, **comb. nov.** [urn:lsid:ipni.org:names:60476507–2] ≡ *Calycosiphonia pentamera* Sonké & Robbr., Nordic J. Bot. 25: 276 (2007 publ. 2008).

*Type*: CAMEROON, 3 km northwest of Mbikiliki, (03°11’24.9”N, 10°31’35.3”E), 21 Jan 2006 (fl.), *B*. *Sonké & K*. *M*.*-N*. *Djuikouo 4320* (holotype: BR; isotypes: BR including flowers in spirit, BRLU, K, MO, WAG, YA).

Distribution: Western Cameroon, Equatorial Guinea, widespread in the evergreen lowland rainforests of eastern Cameroon, extending into Rio Muni. (Fig 4).

**5. *Kupeantha spathulata*** (A.P.Davis & Sonké) Cheek, **comb. nov.** [urn:lsid:ipni.org:names:60476508–2] ≡ *Argocoffeopsis spathulata* A.P.Davis & Sonké, Blumea 53: 528 (2008).

Type: CAMEROON, Mvilé, 3 km NNW Ngovayang (03°13’41”N, 10°34’52”E), 30 Nov 2005, *B*. *Sonké & K*. *M*.*-N*. *Djuikouo 4188* (holotype: K; isotype: BR, BRLU, MO, WAG, YA).

Distribution: Cameroon; relatively widespread in the lowland evergreen forests of eastern Cameroon. (Fig 4).

## Supporting information

S1 Appendix Voucher information and GenBank accession numbers for taxa used in this study. A dash indicates that the region was not sampled. Voucher specimens are deposited in the following herbaria: BR = National Botanic Garden of Belgium, Meise; DSM = University of Dar es Salaam; K = Royal Botanic Gardens, Kew; TAN = Parc de Tsimbazaza, Antananarivo; YA = National Herbarium of Cameroon, Yaoundé.
(DOC)

S1 File Concatenated sequence alignment, with sequences from the plastid markers *rpl16*, *trnL-F* and *accD-psa1*. (FAS)

S1 Fig Best scoring ML tree for the concatenated dataset, with bootstrap values equal or higher than 70% displayed above the branches. (TIF)
