# Supplementary material for: Kupeantha (Coffeeae, Rubiaceae), a new genus from Cameroon and Equatorial Guinea
Source: PLoS One. 2018 Jun 26;13(6):e0199324. doi: 10.1371/journal.pone.0199324 (PMC6019108; doi:10.1371/journal.pone.0199324)
Supplement: S1 Appendix — A dash indicates that the region was not sampled. Voucher specimens are deposited in the following herbaria: BR = National Botanic Garden of Belgium, Meise; DSM = University of Dar es Salaam; K = Royal Botanic Gardens, Kew; TAN = Parc de Tsimbazaza, Antananarivo; YA = National Herbarium of Cameroon, Yaoundé. (DOC) [file pone.0199324.s001.doc]

***Taxon—****Voucher* (herbarium), Origin; GenBank accession nos.: *accD-psaI, rpl16, trnL-F.*

***Argocoffeopsis eketensis*** (Wernham) Robbr.—*Davis 3031* (K), Cameroon; [DQ180497](https://www.ncbi.nlm.nih.gov/nuccore/DQ180497), [DQ180531](https://www.ncbi.nlm.nih.gov/nuccore/DQ180531), [DQ180566](https://www.ncbi.nlm.nih.gov/nuccore/DQ180566). ***Argocoffeopsis rupestris*** (Hiern) Robbr.—*Schmidt 1646* (K), Ghana; [**MG214502**](https://www.ncbi.nlm.nih.gov/nuccore/MG214502), [**MG214509**](https://www.ncbi.nlm.nih.gov/nuccore/MG214509), [**MG214511**](https://www.ncbi.nlm.nih.gov/nuccore/MG214511). ***Argocoffeopsis scandens*** (K.Schum.) Lebrun—*Davis 3016* (K), Cameroon; [DQ180498](https://www.ncbi.nlm.nih.gov/nuccore/DQ180498), [DQ180533](https://www.ncbi.nlm.nih.gov/nuccore/DQ180533), [DQ180568](https://www.ncbi.nlm.nih.gov/nuccore/DQ180568). ***Argocoffeopsis subcordata*** (Hiern) Lebrun—*Luke 12420* (K), Democratic Republic of Congo; [**MG214504**](https://www.ncbi.nlm.nih.gov/nuccore/MG214504), [**MG214507**](https://www.ncbi.nlm.nih.gov/nuccore/MG214507), [**MG214512**](https://www.ncbi.nlm.nih.gov/nuccore/MG214512). ***Argocoffeopsis thonneri*** (Lebrun) Larridon—*Harris 8168* (K), Central African Republic; [DQ180496](https://www.ncbi.nlm.nih.gov/nuccore/DQ180496), [DQ180532](https://www.ncbi.nlm.nih.gov/nuccore/DQ180532), [DQ180567](https://www.ncbi.nlm.nih.gov/nuccore/DQ180567). ***Belonophora coriacea*** Hoyle—*Maurin 5* (K), Cameroon; [DQ180499](https://www.ncbi.nlm.nih.gov/nuccore/DQ180499), [DQ180534](https://www.ncbi.nlm.nih.gov/nuccore/DQ180534), [DQ180569](https://www.ncbi.nlm.nih.gov/nuccore/DQ180569). ***Belonophora coriacea*** Hoyle—*Maurin 19* (K), Cameroon; [DQ180500](https://www.ncbi.nlm.nih.gov/nuccore/DQ180500), [DQ180535](https://www.ncbi.nlm.nih.gov/nuccore/DQ180535), [DQ180570](https://www.ncbi.nlm.nih.gov/nuccore/DQ180570). ***Belonophora*** sp.—*TAF 480* (K), Cameroon; [DQ180501](https://www.ncbi.nlm.nih.gov/nuccore/DQ180501), [DQ180536](https://www.ncbi.nlm.nih.gov/nuccore/DQ180536), [DQ180571](https://www.ncbi.nlm.nih.gov/nuccore/DQ180571). ***Calycosiphonia macrochlamys*** (K.Schum.) Robbr.—*Davis 3036* (K), Cameroon; [DQ180506](https://www.ncbi.nlm.nih.gov/nuccore/DQ180506), [DQ180541](https://www.ncbi.nlm.nih.gov/nuccore/DQ180541), [DQ180575](https://www.ncbi.nlm.nih.gov/nuccore/DQ180575). ***Calycosiphonia macrochlamys*** (K.Schum.) Robbr.—*Davis 3044* (K), Cameroon; [DQ180507](https://www.ncbi.nlm.nih.gov/nuccore/DQ180507), [DQ180542](https://www.ncbi.nlm.nih.gov/nuccore/DQ180542), [DQ180576](https://www.ncbi.nlm.nih.gov/nuccore/DQ180576). ***Calycosiphonia spathicalyx*** (K.Schum.) Robbr.—*Mvungi 22* (DSM, K), Tanzania; [DQ180508](https://www.ncbi.nlm.nih.gov/nuccore/DQ180508), [DQ180543](https://www.ncbi.nlm.nih.gov/nuccore/DQ180543), [DQ180577](https://www.ncbi.nlm.nih.gov/nuccore/DQ180577). ***Calycosiphonia spathicalyx*** (K.Schum.) Robbr.—*Davis 2925* (K), Tanzania; [DQ180509](https://www.ncbi.nlm.nih.gov/nuccore/DQ180509), [DQ180544](https://www.ncbi.nlm.nih.gov/nuccore/DQ180544), [DQ180578](https://www.ncbi.nlm.nih.gov/nuccore/DQ180578). ***Coffea arabica*** L.— *Jaufeerally-Fakim 29* (K), Mascarenes; [DQ153727](https://www.ncbi.nlm.nih.gov/nuccore/DQ153727), [DQ153845](https://www.ncbi.nlm.nih.gov/nuccore/DQ153845), [DQ153478](https://www.ncbi.nlm.nih.gov/nuccore/DQ153478). ***Coffea ebracteolata*** (Hiern) Brenan—*Davis 3008* (K), Cameroon; [DQ153392](https://www.ncbi.nlm.nih.gov/nuccore/DQ153392), [DQ153641](https://www.ncbi.nlm.nih.gov/nuccore/DQ153641), [DQ153759](https://www.ncbi.nlm.nih.gov/nuccore/DQ153759). ***Coffea kapakata*** (A.Chev.) Bridson—*IRD-Montpellier OK* (K), Angola; [DQ153490](https://www.ncbi.nlm.nih.gov/nuccore/DQ153490), [DQ153739](https://www.ncbi.nlm.nih.gov/nuccore/DQ153739), [DQ153857](https://www.ncbi.nlm.nih.gov/nuccore/DQ153857). ***Coffea mannii*** (Hook.f.) A.P.Davis—*Davis 6958* (K), Central African Republic; [DQ180518](https://www.ncbi.nlm.nih.gov/nuccore/DQ180518), [DQ180553](https://www.ncbi.nlm.nih.gov/nuccore/DQ180553), [DQ180587](https://www.ncbi.nlm.nih.gov/nuccore/DQ180587). ***Coffea mangoroensis*** Portères—*Rakotonasolo 41* (K, TAN), Madagascar; [DQ153503](https://www.ncbi.nlm.nih.gov/nuccore/DQ153503), [DQ153752](https://www.ncbi.nlm.nih.gov/nuccore/DQ153752), [DQ153870](https://www.ncbi.nlm.nih.gov/nuccore/DQ153870). ***Coffea moratii*** J.-F.Leroy ex A.P.Davis & Rakotonas.—*Davis 2326* (K), Madagascar; [DQ153502](https://www.ncbi.nlm.nih.gov/nuccore/DQ153502), [DQ153751](https://www.ncbi.nlm.nih.gov/nuccore/DQ153751), [DQ153869](https://www.ncbi.nlm.nih.gov/nuccore/DQ153869). ***Coffea sapinii*** (De Wild.) A.P.Davis—*Sapin s.n. 0856914* (BR), Congo-Kinshasa; [DQ153394](https://www.ncbi.nlm.nih.gov/nuccore/DQ153394), [DQ153643](https://www.ncbi.nlm.nih.gov/nuccore/DQ153643), [DQ153761](https://www.ncbi.nlm.nih.gov/nuccore/DQ153761). ***Coffea semsei*** (Bridson) A.P.Davis—*Kisera 1473* (K), Tanzania; [DQ153395](https://www.ncbi.nlm.nih.gov/nuccore/DQ153395), [DQ153644](https://www.ncbi.nlm.nih.gov/nuccore/DQ153644), [DQ153762](https://www.ncbi.nlm.nih.gov/nuccore/DQ153762). ***Coffea sessiliflora*** Bridson— *Mvungi 25* (K, DSM), Tanzania; DQ153700, DQ153818, DQ15345. ***Diplospora dubia*** (Lindl.) Masam.—*Van Caekenberghe 49* (BR); [AM999388](https://www.ncbi.nlm.nih.gov/nuccore/AM999388.1), [AM999526](https://www.ncbi.nlm.nih.gov/nuccore/AM999526.1), [AM999468](https://www.ncbi.nlm.nih.gov/nuccore/AM999468.1). ***Diplospora*** sp.—*Bremer 15238* (K), Borneo (Brunei); [DQ180511](https://www.ncbi.nlm.nih.gov/nuccore/DQ180511), [DQ180546](https://www.ncbi.nlm.nih.gov/nuccore/DQ180546), [DQ180580](https://www.ncbi.nlm.nih.gov/nuccore/DQ180580). ***Discospermum abnorme*** (Korth.) S.J.Ali & Robbr.—*DB 87* (K), Borneo; [DQ180512](https://www.ncbi.nlm.nih.gov/nuccore/DQ180512), [DQ180547](https://www.ncbi.nlm.nih.gov/nuccore/DQ180547), [DQ180581](https://www.ncbi.nlm.nih.gov/nuccore/DQ180581). ***Empogona gossweileri*** (S.Moore) Tosh & Robbr.—*Senterre 4041* (K), Equatorial Guinea; [AM999365](https://www.ncbi.nlm.nih.gov/nuccore/AM999365.1), [FM160598](https://www.ncbi.nlm.nih.gov/nuccore/FM160598.1), [AM999492](https://www.ncbi.nlm.nih.gov/nuccore/AM999492.1). ***Empogona lanceolata*** (Sond.) Tosh & Robbr.—*Bagliss 1519* (K), South Africa; [AM999370](https://www.ncbi.nlm.nih.gov/nuccore/AM999370.1), [FM160603](https://www.ncbi.nlm.nih.gov/nuccore/FM160603.1), [AM999497](https://www.ncbi.nlm.nih.gov/nuccore/AM999497.1). ***Empogona ngalaensis*** (Robbr.) Tosh & Robbr.—*Bidgood 2966* (K), Tanzania; [AM999374](https://www.ncbi.nlm.nih.gov/nuccore/AM999374.1), [FM160607](https://www.ncbi.nlm.nih.gov/nuccore/FM160607.1), [AM999501](https://www.ncbi.nlm.nih.gov/nuccore/AM999501.1). ***Empogona talbotii*** (Wernham) Tosh & Robbr.—*Latilo 67674* (K), Nigeria; [AM999386](https://www.ncbi.nlm.nih.gov/nuccore/AM999386.1), [FM160618](https://www.ncbi.nlm.nih.gov/nuccore/FM160618.1), [AM999509](https://www.ncbi.nlm.nih.gov/nuccore/AM999509.1). ***Gardenia thunbergia*** L. f.—*Davis et al. 1961–29703* (K), SE Africa; [DQ180514](https://www.ncbi.nlm.nih.gov/nuccore/DQ180514), [DQ180549](https://www.ncbi.nlm.nih.gov/nuccore/DQ180549), [DQ180583](https://www.ncbi.nlm.nih.gov/nuccore/DQ180583). ***Kupeantha ebo*** M.Alvarez & Cheek—Alvarez 11 (K), Cameroon; [**MG214505**](https://www.ncbi.nlm.nih.gov/nuccore/MG214505), [**MG214508**](https://www.ncbi.nlm.nih.gov/nuccore/MG214508), [**MG214513**](https://www.ncbi.nlm.nih.gov/nuccore/MG214513). ***Kupeantha fosimondi*** (Tchiengué & Cheek) Cheek (*Argocoffeopsis fosimondi* Tchiengué & Cheek)—Tchiengué 2213 (K), Cameroon; [**MG214506**](https://www.ncbi.nlm.nih.gov/nuccore/MG214506), - , [**MG214515**](https://www.ncbi.nlm.nih.gov/nuccore/MG214515). ***Kupeantha kupensis*** Cheek & Sonké—Cheek 7882 (K), Cameroon; [**MG214503**](https://www.ncbi.nlm.nih.gov/nuccore/MG214503), [**MG214510**](https://www.ncbi.nlm.nih.gov/nuccore/MG214510), [**MG214514**](https://www.ncbi.nlm.nih.gov/nuccore/MG214514). ***Kupeantha* *spathulata*** (A.P.Davis & Sonké) Cheek (*Argocoffeopsis* *spathulata* A.P.Davis & Sonké)—*Sonké 3783* (K, YA), Cameroon; [DQ180505](https://www.ncbi.nlm.nih.gov/nuccore/DQ180505), [DQ180540](https://www.ncbi.nlm.nih.gov/nuccore/DQ180540), [DQ180565](https://www.ncbi.nlm.nih.gov/nuccore/DQ180565). ***Sericanthe andongensis*** (Hiern) Robbr.—*Bidgood 3490* (K), Tanzania; [DQ180522](https://www.ncbi.nlm.nih.gov/nuccore/DQ180522), [DQ180557](https://www.ncbi.nlm.nih.gov/nuccore/DQ180557), [DQ180591](https://www.ncbi.nlm.nih.gov/nuccore/DQ180591). ***Sericanthe jacfelicis*** (N.Hallé) Robbr.—*Carvalho 4169* (K), Gulf of Guinea Islands (Bioko); [DQ180523](https://www.ncbi.nlm.nih.gov/nuccore/DQ180523.1), -, [DQ180592](https://www.ncbi.nlm.nih.gov/nuccore/DQ180592.1). ***Tricalysia anomala*** E.A.Bruce var. ***guineensis*** Robbr.—*Davis 3045* (K), Cameroon; [DQ180526](https://www.ncbi.nlm.nih.gov/nuccore/DQ180526), [DQ180560](https://www.ncbi.nlm.nih.gov/nuccore/DQ180560), [DQ180595](https://www.ncbi.nlm.nih.gov/nuccore/DQ180595). ***Tricalysia cryptocalyx*** Baker—*Davis 2173* (K), Madagascar; [DQ153400](https://www.ncbi.nlm.nih.gov/nuccore/DQ153400), [DQ153649](https://www.ncbi.nlm.nih.gov/nuccore/DQ153649), [DQ153767](https://www.ncbi.nlm.nih.gov/nuccore/DQ153767). ***Tricalysia elliottii*** (K.Schum.) Hutch. & Dalziel—*Jongkind 1806* (K), Ghana; [AM999364](https://www.ncbi.nlm.nih.gov/nuccore/AM999364.1), [FM160597](https://www.ncbi.nlm.nih.gov/nuccore/FM160597), [AM999491](https://www.ncbi.nlm.nih.gov/nuccore/AM999491.1). ***Tricalysia jasminiflora*** (Klotzsch) Benth. & Hook.f. ex Hiern—*Ayami 42* (K), Malawi; [AM999368](https://www.ncbi.nlm.nih.gov/nuccore/AM999368.1), [FM160601](https://www.ncbi.nlm.nih.gov/nuccore/FM160601.1), [AM999495](https://www.ncbi.nlm.nih.gov/nuccore/AM999495.1). ***Tricalysia leucocarpa*** (Baill.) Ranariv. & De Block—*Gautier 2442* (K), Madagascar; [AM999371](https://www.ncbi.nlm.nih.gov/nuccore/AM999371.1), [FM160604](https://www.ncbi.nlm.nih.gov/nuccore/FM160604.1), [AM999498](https://www.ncbi.nlm.nih.gov/nuccore/AM999498.1). ***Tricalysia pallens*** Hiern—*Adams 831* (K), Liberia; [AM999379](https://www.ncbi.nlm.nih.gov/nuccore/AM999379.1), [FM160612](https://www.ncbi.nlm.nih.gov/nuccore/FM160612.1), [AM999506](https://www.ncbi.nlm.nih.gov/nuccore/AM999506.1). ***Tricalysia schliebenii*** Robbr.—*Bidgood 1913* (K), Tanzania; [AM999385](https://www.ncbi.nlm.nih.gov/nuccore/AM999385.1), [FM160617](https://www.ncbi.nlm.nih.gov/nuccore/FM160617.1), [AM999508](https://www.ncbi.nlm.nih.gov/nuccore/AM999508.1). ***Xantonnea parvifolia*** (Kuntze) Craib—*Cantaranothai 895* (K), Thailand; [DQ180530](https://www.ncbi.nlm.nih.gov/nuccore/DQ180530), [DQ180564](https://www.ncbi.nlm.nih.gov/nuccore/DQ180564), [DQ180599](https://www.ncbi.nlm.nih.gov/nuccore/DQ180599).
