# Supplementary figures and images for: Kupeantha (Coffeeae, Rubiaceae), a new genus from Cameroon and Equatorial Guinea
Source: PLoS One. 2018 Jun 26;13(6):e0199324. doi: 10.1371/journal.pone.0199324 (PMC6019108; doi:10.1371/journal.pone.0199324)

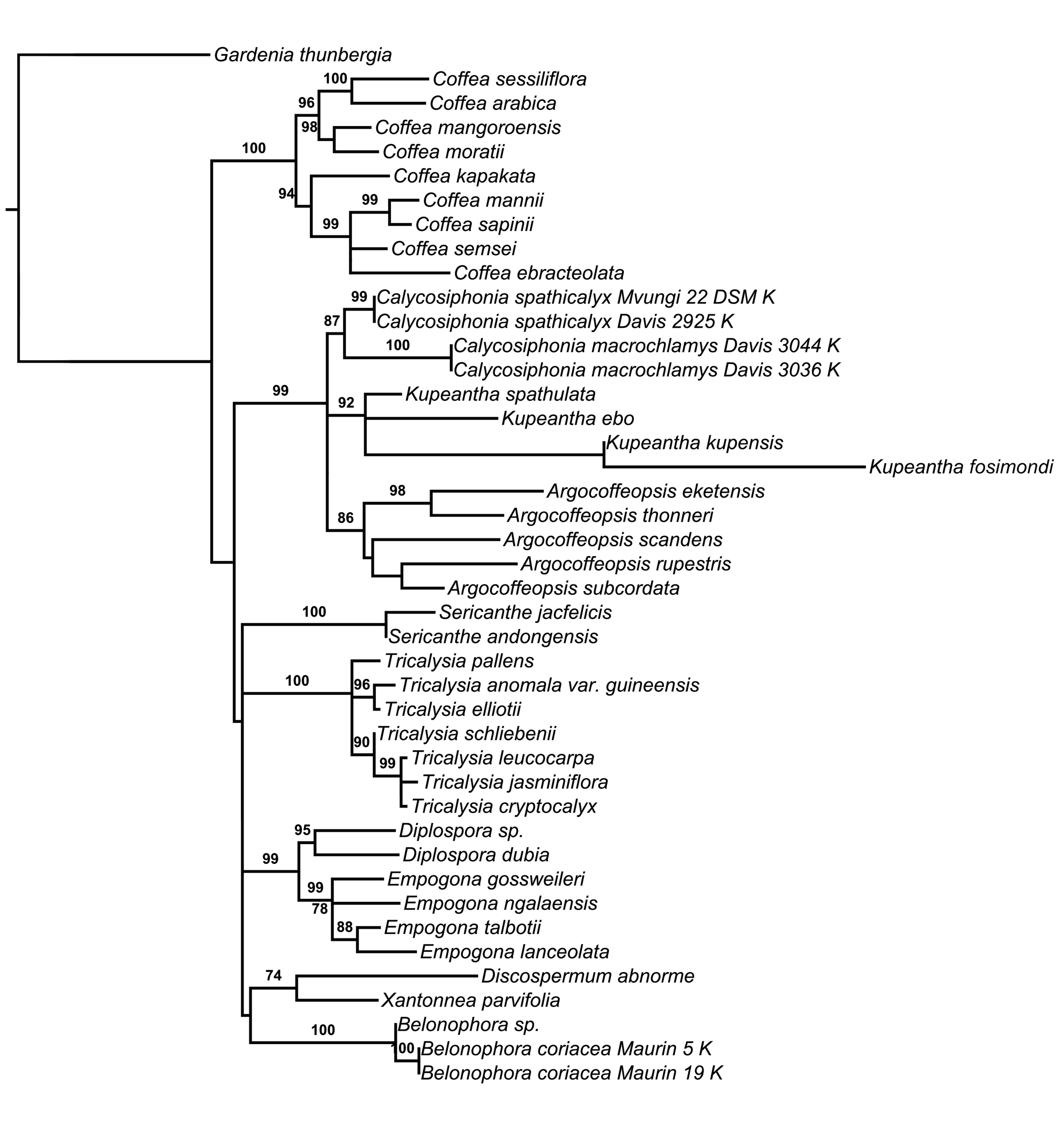

Supplement: S1 Fig — (TIF) [file pone.0199324.s003.tif]
